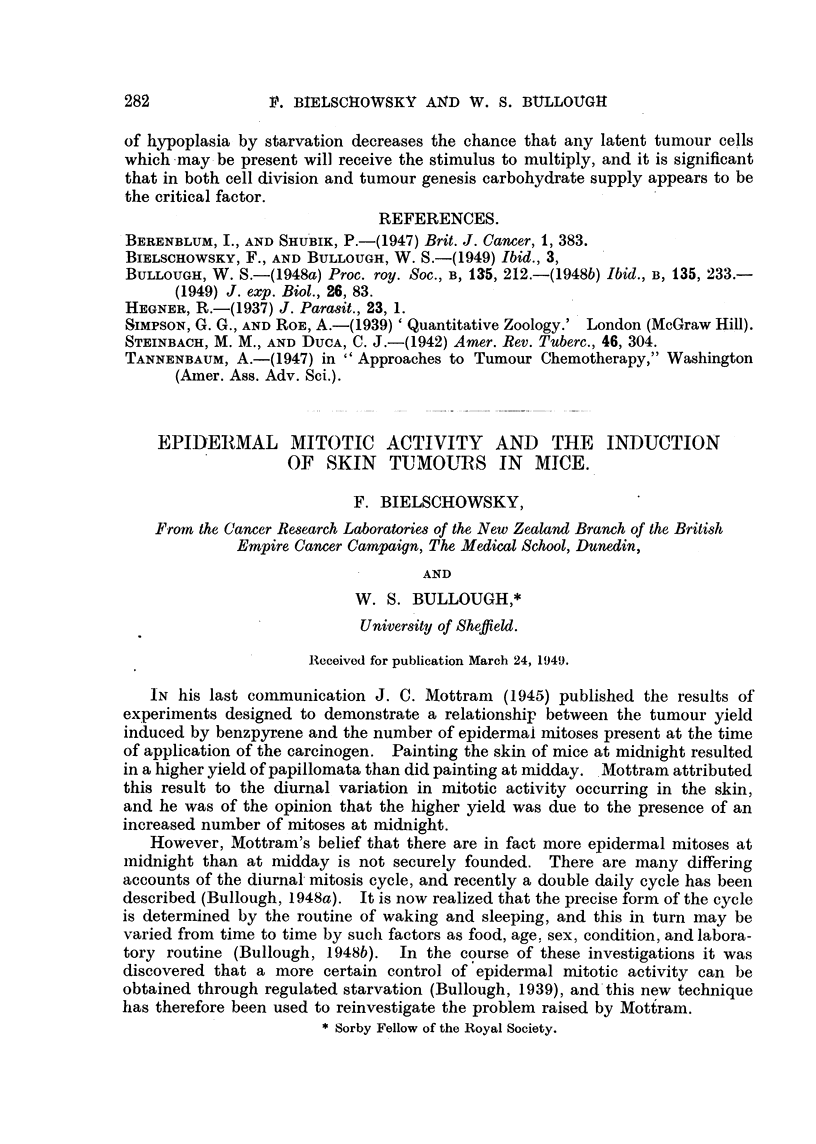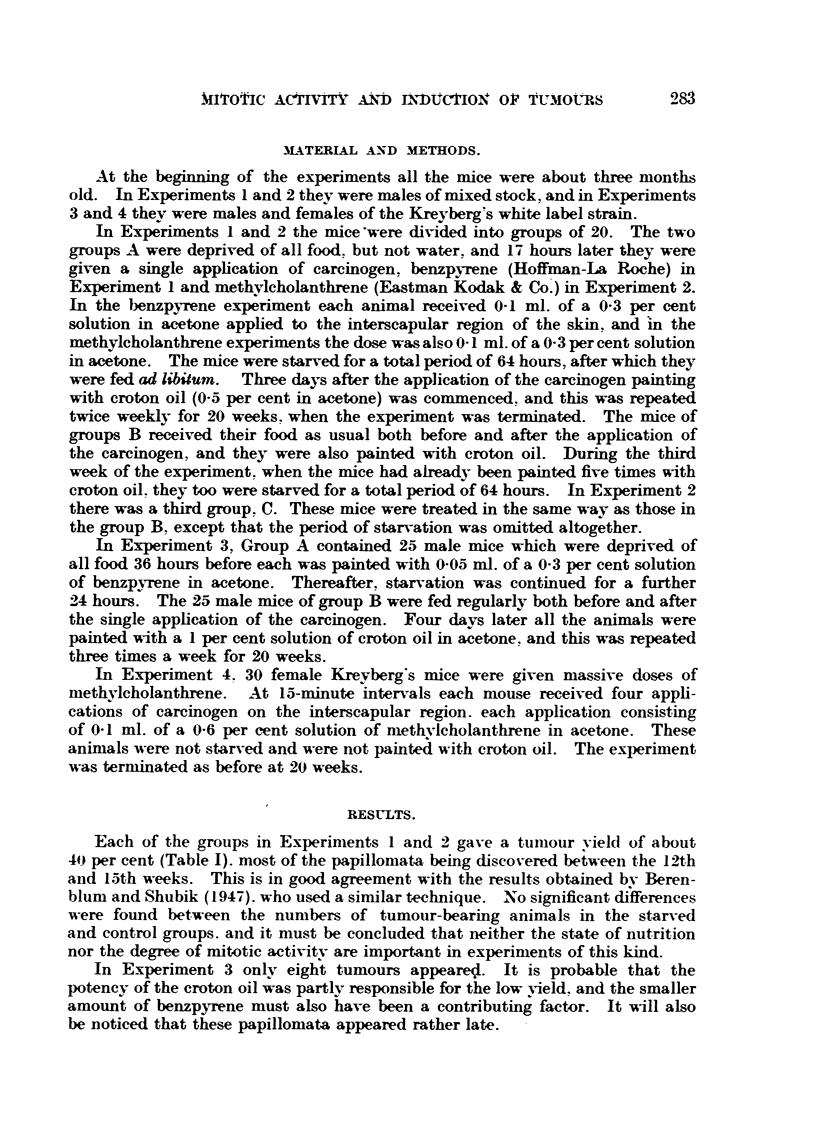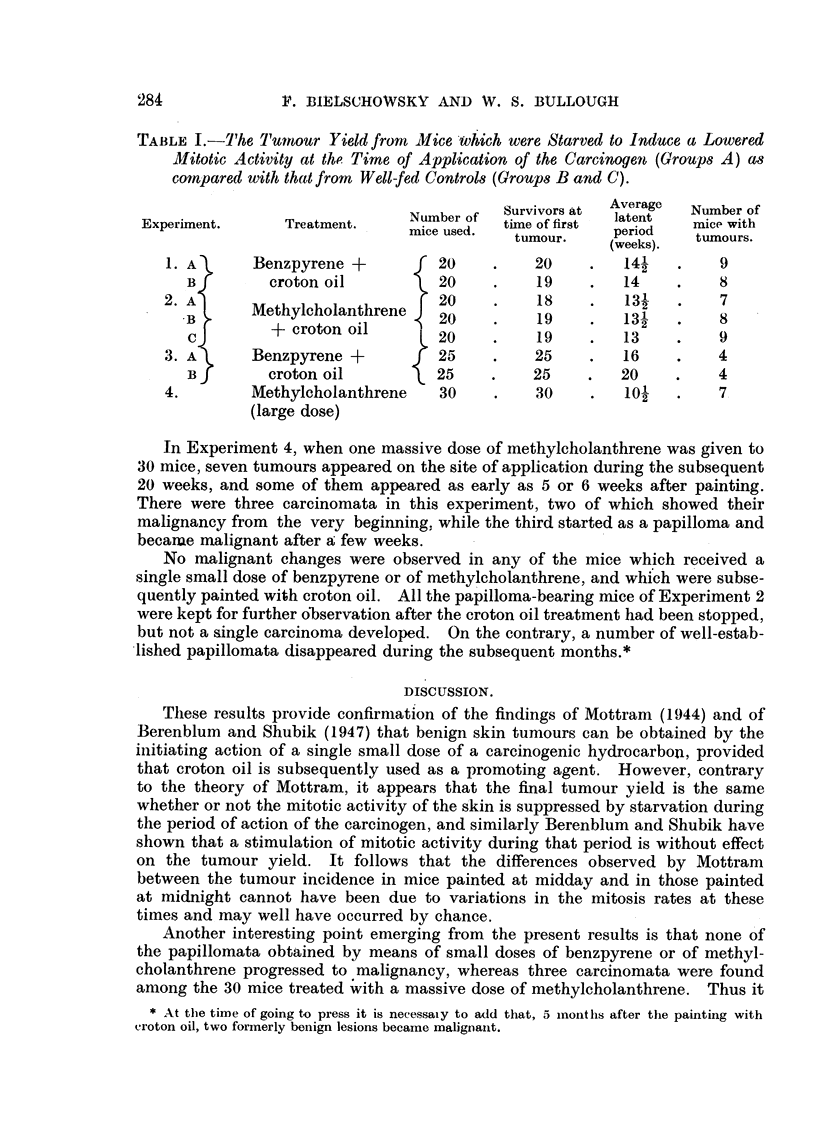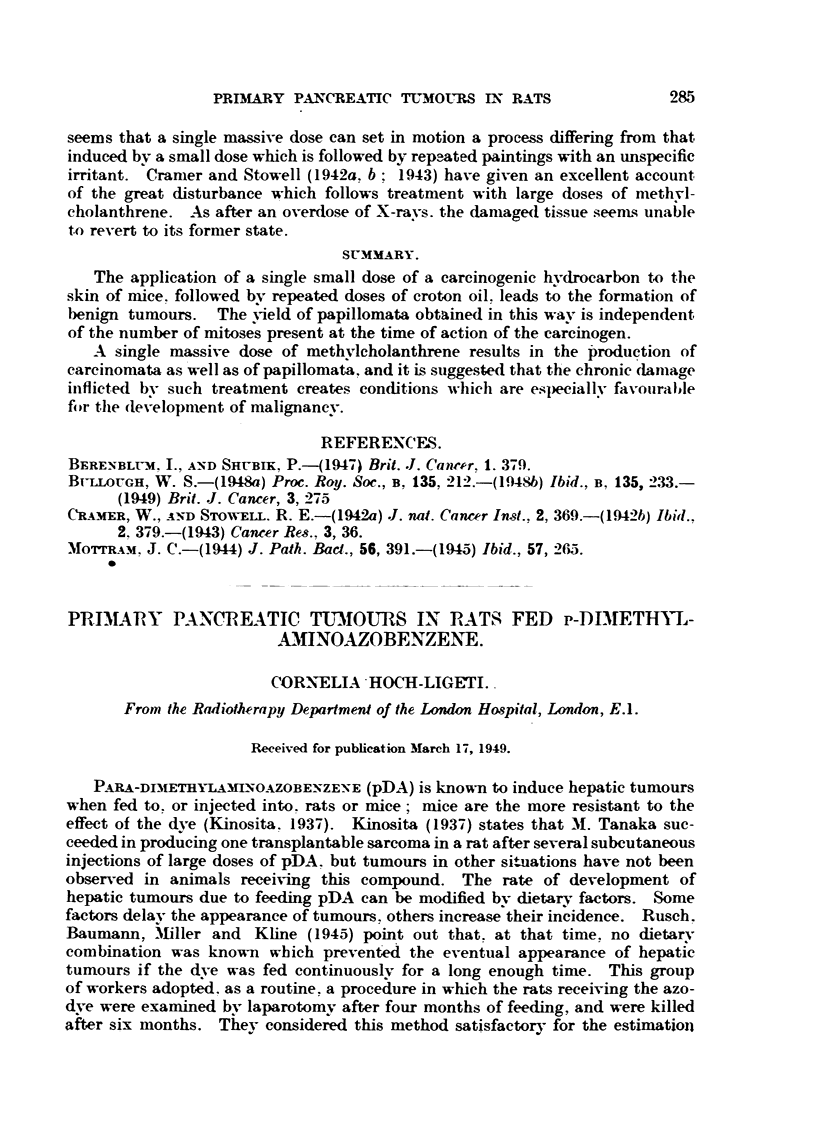# Epidermal Mitotic Activity and the Induction of Skin Tumours in Mice

**DOI:** 10.1038/bjc.1949.32

**Published:** 1949-06

**Authors:** F. Bielschowsky, W. S. Bullough


### EPIDERMAL MITOTIC ACTIVITY AND THE INDUCTION OF SKIN TUMOURS IN MICE.

F. BIELSCHOWSKY,

*From the Cancer Research Laboratories of the New Zealand Branch of the British Empire Cancer Campaign, The Medical School, Dunedin,*

AND

W. S. BULLOUGH,*

*University of Sheffield.*

Received for publication March 24, 1949.

IN his last communication J. C. Mottram (1945) published the results of experiments designed to demonstrate a relationship between the tumour yield induced by benzpyrene and the number of epidermal mitoses present at the time of application of the carcinogen. Painting the skin of mice at midnight resulted in a higher yield of papillomata than did painting at midday. Mottram attributed this result to the diurnal variation in mitotic activity occurring in the skin, and he was of the opinion that the higher yield was due to the presence of an increased number of mitoses at midnight.

However, Mottram's belief that there are in fact more epidermal mitoses at midnight than at midday is not securely founded. There are many differing accounts of the diurnal mitosis cycle, and recently a double daily cycle has been described (Bullough, 1948*a*). It is now realized that the precise form of the cycle is determined by the routine of waking and sleeping, and this in turn may be varied from time to time by such factors as food, age, sex, condition, and laboratory routine (Bullough, 1948*b*). In the course of these investigations it was discovered that a more certain control of epidermal mitotic activity can be obtained through regulated starvation (Bullough, 1939), and this new technique has therefore been used to reinvestigate the problem raised by Mottram.

* Sorby Fellow of the Royal Society.

### MATERIAL AND METHODS.

At the beginning of the experiments all the mice were about three months old. In Experiments 1 and 2 they were males of mixed stock, and in Experiments 3 and 4 they were males and females of the Kreyberg's white label strain.

In Experiments 1 and 2 the mice were divided into groups of 20. The two groups A were deprived of all food, but not water, and 17 hours later they were given a single application of carcinogen, benzpyrene (Hoffman-La Roche) in Experiment 1 and methylcholanthrene (Eastman Kodak & Co.) in Experiment 2. In the benzpyrene experiment each animal received 0·1 ml. of a 0·3 per cent solution in acetone applied to the interscapular region of the skin, and in the methylcholanthrene experiments the dose was also 0·1 ml. of a 0·3 per cent solution in acetone. The mice were starved for a total period of 64 hours, after which they were fed *ad libitum*. Three days after the application of the carcinogen painting with croton oil (0·5 per cent in acetone) was commenced, and this was repeated twice weekly for 20 weeks, when the experiment was terminated. The mice of groups B received their food as usual both before and after the application of the carcinogen, and they were also painted with croton oil. During the third week of the experiment, when the mice had already been painted five times with croton oil, they too were starved for a total period of 64 hours. In Experiment 2 there was a third group, C. These mice were treated in the same way as those in the group B, except that the period of starvation was omitted altogether.

In Experiment 3, Group A contained 25 male mice which were deprived of all food 36 hours before each was painted with 0·05 ml. of a 0·3 per cent solution of benzpyrene in acetone. Thereafter, starvation was continued for a further 24 hours. The 25 male mice of group B were fed regularly both before and after the single application of the carcinogen. Four days later all the animals were painted with a 1 per cent solution of croton oil in acetone, and this was repeated three times a week for 20 weeks.

In Experiment 4, 30 female Kreyberg's mice were given massive doses of methylcholanthrene. At 15-minute intervals each mouse received four applications of carcinogen on the interscapular region, each application consisting of 0·1 ml. of a 0·6 per cent solution of methylcholanthrene in acetone. These animals were not starved and were not painted with croton oil. The experiment was terminated as before at 20 weeks.

### RESULTS.

Each of the groups in Experiments 1 and 2 gave a tumour yield of about 40 per cent (Table I), most of the papillomata being discovered between the 12th and 15th weeks. This is in good agreement with the results obtained by Berenblum and Shubik (1947), who used a similar technique. No significant differences were found between the numbers of tumour-bearing animals in the starved and control groups, and it must be concluded that neither the state of nutrition nor the degree of mitotic activity are important in experiments of this kind.

In Experiment 3 only eight tumours appeared. It is probable that the potency of the croton oil was partly responsible for the low yield, and the smaller amount of benzpyrene must also have been a contributing factor. It will also be noticed that these papillomata appeared rather late.

TABLE I.—*The Tumour Yield from Mice which were Starved to Induce a Lowered Mitotic Activity at the Time of Application of the Carcinogen (Groups A) as compared with that from Well-fed Controls (Groups B and C).*

| Experiment. | Treatment. | Number of mice used. | Survivors at time of first tumour. | Average latent period (weeks). | Number of mice with tumours. |
|---|---|---|---|---|---|
| 1. A | Benzpyrene + croton oil | 20 | 20 | 14½ | 9 |
| B | | 20 | 19 | 14 | 8 |
| 2. A | Methylcholanthrene + croton oil | 20 | 18 | 13½ | 7 |
| B | | 20 | 19 | 13½ | 8 |
| C | | 20 | 19 | 13 | 9 |
| 3. A | Benzpyrene + croton oil | 25 | 25 | 16 | 4 |
| B | | 25 | 25 | 20 | 4 |
| 4. | Methylcholanthrene (large dose) | 30 | 30 | 10½ | 7 |

In Experiment 4, when one massive dose of methylcholanthrene was given to 30 mice, seven tumours appeared on the site of application during the subsequent 20 weeks, and some of them appeared as early as 5 or 6 weeks after painting. There were three carcinomata in this experiment, two of which showed their malignancy from the very beginning, while the third started as a papilloma and became malignant after a few weeks.

No malignant changes were observed in any of the mice which received a single small dose of benzpyrene or of methylcholanthrene, and which were subsequently painted with croton oil. All the papilloma-bearing mice of Experiment 2 were kept for further observation after the croton oil treatment had been stopped, but not a single carcinoma developed. On the contrary, a number of well-established papillomata disappeared during the subsequent months.*

### DISCUSSION.

These results provide confirmation of the findings of Mottram (1944) and of Berenblum and Shubik (1947) that benign skin tumours can be obtained by the initiating action of a single small dose of a carcinogenic hydrocarbon, provided that croton oil is subsequently used as a promoting agent. However, contrary to the theory of Mottram, it appears that the final tumour yield is the same whether or not the mitotic activity of the skin is suppressed by starvation during the period of action of the carcinogen, and similarly Berenblum and Shubik have shown that a stimulation of mitotic activity during that period is without effect on the tumour yield. It follows that the differences observed by Mottram between the tumour incidence in mice painted at midday and in those painted at midnight cannot have been due to variations in the mitosis rates at these times and may well have occurred by chance.

Another interesting point emerging from the present results is that none of the papillomata obtained by means of small doses of benzpyrene or of methylcholanthrene progressed to malignancy, whereas three carcinomata were found among the 30 mice treated with a massive dose of methylcholanthrene. Thus it

* At the time of going to press it is necessary to add that, 5 months after the painting with croton oil, two formerly benign lesions became malignant.

seems that a single massive dose can set in motion a process differing from that induced by a small dose which is followed by repeated paintings with an unspecific irritant. Cramer and Stowell (1942*a*, *b* ; 1943) have given an excellent account of the great disturbance which follows treatment with large doses of methylcholanthrene. As after an overdose of X-rays, the damaged tissue seems unable to revert to its former state.

SUMMARY.

The application of a single small dose of a carcinogenic hydrocarbon to the skin of mice, followed by repeated doses of croton oil, leads to the formation of benign tumours. The yield of papillomata obtained in this way is independent of the number of mitoses present at the time of action of the carcinogen.

A single massive dose of methylcholanthrene results in the production of carcinomata as well as of papillomata, and it is suggested that the chronic damage inflicted by such treatment creates conditions which are especially favourable for the development of malignancy.

### REFERENCES.

BERENBLUM, I., AND SHUBIK, P.—(1947) *Brit. J. Cancer*, **1**, 379.

BULLOUGH, W. S.—(1948*a*) *Proc. Roy. Soc.*, B, **135**, 212.—(1948*b*) *Ibid.*, B, **135**, 233.—(1949) *Brit. J. Cancer*, **3**, 275

CRAMER, W., AND STOWELL, R. E.—(1942*a*) *J. nat. Cancer Inst.*, **2**, 369.—(1942*b*) *Ibid.*, **2**, 379.—(1943) *Cancer Res.*, **3**, 36.

MOTTRAM, J. C.—(1944) *J. Path. Bact.*, **56**, 391.—(1945) *Ibid.*, **57**, 265.